# Epidemiology and Outcomes of Clostridial Bacteremia at a Tertiary-Care Institution

**DOI:** 10.1100/tsw.2009.21

**Published:** 2009-02-28

**Authors:** Monica Shah, Eliahu Bishburg, David A. Baran, Trini Chan

**Affiliations:** Newark Beth Israel Medical Center, Newark, USA

**Keywords:** Clostridium, bacteremia, mortality, anaerobe, antibiotics

## Abstract

Clostridial bacteremia (CB) is a rare clinical entity, accounting for less than 2-3% of all blood cultures. CB is frequently associated with intra-abdominal infections and underlying malignancy, particularly colon cancer or leukemia. *Clostridium* species are commonly isolated from blood cultures as a part of polymicrobial bacteremia. The mortality rate among patients with CB has been reported to be as high as 50%. The presentation and outcome of CB depends on underlying host defenses and the type of *Clostridium* species causing infection. A favorable outcome for CB appears to depend on the prompt initiation of appropriate antibiotics and surgical intervention. All patients with positive blood cultures for *Clostridium* species, from January 1995 to December 2003, were included in this study. Medical records of these patients were reviewed for age, sex, underlying diseases (such as malignancy and diabetes), antibiotic use, and outcome. Antimicrobial therapy was defined as either “appropriate” or “insufficient” based on its activity against *Clostridium* species. In-hospital, postdiagnosis survival was examined by Kaplan-Meier methodology and comparisons made by the Mantel-Cox Log-Rank test. Ninety-two percent of the patients had monomicrobial CB. C. perfringens was the most frequently isolated pathogen, seen in 60% of cases. The most common underlying conditions were genitourinary and gastrointestinal malignancies, and diabetes. The overall mortality was 48%. Patients with malignancy had a significantly higher 2-day mortality rate (54%) compared to patients without malignancy (8%, p = 0.023). The mortality rates varied according to type of *Clostridium* species. Patients with C. innocuum bacteremia had a significantly higher 2-day mortality rate (100%) compared to patients with C. septicum (67%), and to patients with C. perfringens (27%) (p = 0.004). “appropriate” antibiotics were given to 64% of the patients, 16% were on antibiotics with “insufficient” coverage, and 20% were not given any antibiotics. Patients receiving “insufficient” antibiotic therapy had a significantly higher 2-day mortality rate (75%) compared to patients on “appropriate” antibiotics for *Clostridium* (12.5%) (p = 0.011). CB is associated with high and rapid mortality, especially in patients with malignancy. Early mortality was significantly lower in patients receiving antibiotics with adequate coverage for *Clostridium* species.

*This study was presented in part as a poster presentation at the 44th Interscience Conference on Antimicrobial Agents and Chemotherapy (ICAAC). Washington, D.C., October 30–November 2, 2004.
**Corresponding author.
©2009 with author.
Published by TheScientificWorld; www.thescientificworld.com

## INTRODUCTION

The genus *Clostridium* is a group of anaerobic, Gram-positive, spore-forming bacteria. Clostridial bacteremia (CB) is associated primarily with intra-abdominal sepsis due to trauma or surgery, or may occur in patients with malignancies or diabetes[1]. CB is uncommon among hospitalized patients, representing less than 2% of positive blood cultures, and is predominantly seen as part of polymicrobial bacteremia[2]. CB is associated with a high mortality[3], and is usually rapidly fatal without prompt and appropriate antibiotic therapy[4,5]. The outcome of CB depends on the type of *Clostridium* species and the status of the host[4].

In order to determine the epidemiology and incidence of polymicrobial vs. monomicrobial CB, the rate of appropriate antimicrobial therapy for CB, and the association of outcome with different clostridial species, we performed a review at our tertiary-care, teaching hospital.

## PATIENTS AND METHODS

All patients with blood cultures positive for *Clostridium* species, from January 1995 to December 2003, were included. Medical records of these patients were reviewed for age, sex, underlying diseases (such as malignancy and diabetes), antibiotic use, and outcome. *Clostridium* isolates were identified by the microbiology laboratory using the Anaerobic API System (Biomerieux, Durham, NC). The study was approved by the institutional review board.

Antimicrobial therapy was defined as either "appropriate" or "insufficient" based on its activity against *Clostridium* species. Penicillin G, clindamycin, cefoxitin, metronidazole, ampicillin/sulbactam, piperacillin/tazobactam, and imipenem/cilastatin were considered "appropriate" therapy[6]. Other antibiotic regimens were classified as "insufficient" due to their lack of activity against *Clostridium* species.

In-hospital, postdiagnosis survival was examined by Kaplan-Meier methodology and comparisons made by the Mantel-Cox Log-Rank test.

## RESULTS

The blood cultures of 25 patients yielded *Clostridium* species. The mean age was 64 years (SD ± 16.0). Eleven patients (44%) were male.

The underlying conditions of patients with CB were malignancy in 13 (52%), diabetes in 10 (40%), end-stage liver disease in two (8%), and recent cesarean section in one (4%). Of the 13 patients with underlying malignancies, five (38.5%) had genitourinary malignancy, three (23.1%) gastrointestinal, two (15.4%) hematologic, two (15.4%) lung, and one (7.7%) breast cancer.

Of the 25 *Clostridium* isolates, *C. perfringens* was the most frequent (60%), followed by *Clostridium* species-not-further-identified (16%), *C. septicum* (12%), *C. innocuum* (8%), and *C. sordellii* (4%). Five patients had one other organism in addition to the *Clostridium* species: *Bacteroides fragilis* (one patient), *Klebsiella pneumoniae* (one patient), *Staphylococcus epidermidis* (one patient), *S. capitis* (one patient) and *S. simulans* (one patient). The three coagulase-negative *Staphylocccus* isolates were considered contaminants. Polymicrobial CB was seen in two (8%) patients and monomicrobial CB in 23 (92%) patients.

"Appropriate" antibiotics were given to 16 patients (64%); four (16%) were on antibiotics with "insufficient" coverage, five (20%) were not given any antibiotics. Of the patients who received "appropriate" antibiotics, the most commonly used agents were penicillin G, clindamycin, metronidazole, and piperacillin/tazobactam.

The overall mortality was 48% (12/25). The mortality rates at days 1, 2, 7, and 28 were 24, 32, 32, and 59.2%, respectively. The maximal risk of death occurred within 2 days of blood cultures being drawn.

Patients with *C. innocuum* bacteremia had a significantly higher 2-day mortality rate (100%) compared to patients with *C. septicum* (67%) and to patients with *C. perfringens* (27%); $p = 0.004$ by log-rank (Fig. 1). None of the patients with bacteremia due to *C. sordellii* and unspeciated *Clostridium* isolates expired. Patients with malignancy had a significantly higher 2-day mortality rate (54%) compared to patients without malignancy (8%) ($p = 0.023$). Patients receiving "insufficient" antibiotic therapy had a significantly higher 2-day mortality rate (75%) compared to patients on "appropriate" antibiotics for *Clostridium* (12.5%) ($p = 0.011$).

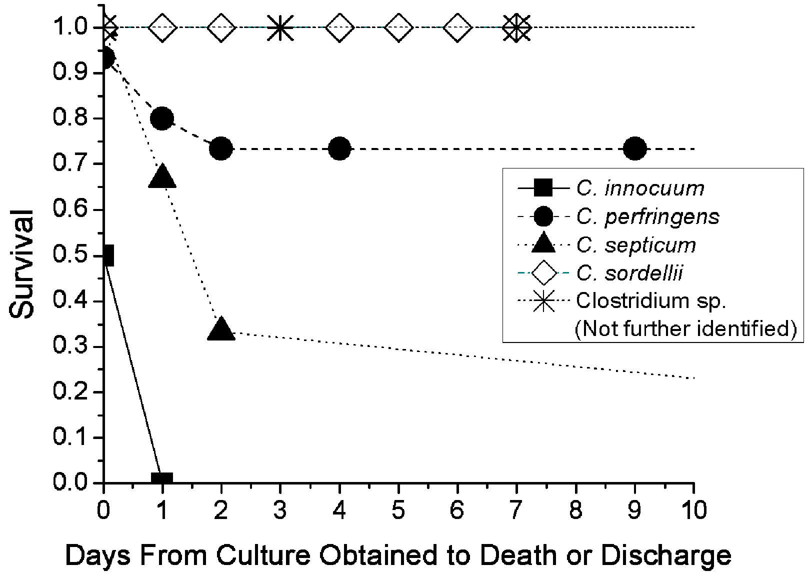

**FIGURE 1.** Kaplan-Meier survival plot of patients with CB vs. days after blood cultures were obtained. Note steep drop in survival at 2 days.

## DISCUSSION

*Clostridium* species have been reported to be a part of polymicrobial bacteremia. In our study, 92% of patients had monomicrobial CB and only 8% had polymicrobial CB. Other studies have reported higher rates of polymicrobial CB, ranging from 33 to 39%[4,5,7].

In our study, the most common underlying conditions in patients with CB were genitourinary and gastrointestinal malignancies, and diabetes. Similar findings have been reported[1,2,5,8]. In a recently published study of CB conducted in a large Canadian health region, a somewhat unexpected finding was that hemodialysis patients were at a more than 200-fold increased risk for development of CB compared to the general population[8]. The study authors believed that hemodialysis patients may be at increased risk due to the high incidence of other comorbid conditions and their regular need for invasive access.

Our finding that the overall mortality rate in patients with CB was 48% corroborates those of previous studies, which have reported overall mortality rates of 41–48%[1,2]. Previous reviews have demonstrated that mortality rates vary with different *Clostridium* species. Myers et al. showed that the mortality rate associated with bacteremia due to *C. septicum* was significantly higher than that with *C. perfringens* or other clostridial species[9]. Our findings show that patients with *C. innocuum* bacteremia had a significantly higher 2-day mortality rate (100%) compared to patients with *C. septicum* (67%) and *C. perfringens* (27%) bacteremia. Both of our patients with *C. innocuum* bacteremia had genitourinary malignancies; one had prostate cancer and the other had renal cell carcinoma. Whether this finding is a coincidence or there is an association between *C. innocuum* and genitourinary tract malignancies warrants further investigation. A previous study reported that four patients with *C. innocuum* bacteremia all survived and the authors felt that the pathogenicity of *C. innocuum* in these patients was uncertain[5]. Contrary to our findings, Leal et al. found no significant associations with different *Clostridium* species and mortality[8].

In our study, patients receiving antibiotics with “insufficient” coverage for *Clostridium* had a significantly higher 2-day mortality rate (75%) compared to patients on “appropriate” antibiotics for *Clostridium* (12.5%). Similarly, in a previous study of patients with CB, the mortality rate was higher (63%) in patients who had not received adequate antibiotic therapy compared to 14% in those who had received adequate antibiotics[10]. Adequate antibiotic coverage for *Clostridium* was the only factor associated with a higher survival rate in this study[10]. Other published reviews have shown that the appropriateness of antibiotic therapy did not significantly affect survival in patients with CB[3,7].

Our study has a few limitations. Our findings are based on data gathered in a retrospective study design; data collection depended on the completeness and accuracy of the patient records. The study was performed in a single institution and has a relatively small sample size. These factors may preclude our data from being applied to other settings. Of the total 25 *Clostridium* bloodstream isolates, four isolates could not be further speciated by the microbiology laboratory.

We assumed that all of the bloodstream isolates of *Clostridium* species were clinically significant. Few studies performed in previous decades have suggested that the isolates of *Clostridium* species from blood cultures often represented contamination or transient bacteremia of little or no clinical significance[11]. However, recent reports have concluded that the growth of *Clostridium* species in blood cultures is often of clinical significance, and that such findings should be appropriately evaluated[8,12].

In conclusion, CB is associated with high and rapid mortality, especially in patients with malignancy. Early mortality was significantly lower in patients receiving antibiotics with adequate coverage for *Clostridium* species. Our study findings suggest that appropriate antibiotics should be initiated promptly in patients presumed to be at risk for CB.
